# Dynamic gaze processing: robust N170 for direct gaze and task-dependent N2pc effects

**DOI:** 10.1093/scan/nsag038

**Published:** 2026-05-22

**Authors:** Nicolas Burra, Océane Lauret

**Affiliations:** Faculté de Psychologie et des Sciences de l’Éducation, Université de Genève, 1205 Geneva, Switzerland; Faculté de Psychologie et des Sciences de l’Éducation, Université de Genève, 1205 Geneva, Switzerland

**Keywords:** N2pc, gaze perception, social relevance, EEG, attention

## Abstract

Eye gaze is a central cue for human social interaction, yet it remains debated whether its effects on perception and attention are automatic or shaped by context. Using EEG across three experiments, we examined how dynamic gaze influences early perceptual encoding (N170) and later attentional selection (N2pc). Participants viewed pairs of faces whose eyes shifted either toward them (gaze toward the observer) or further away (gaze away) while the other closed the eyes. The relevance of eye contact varied across tasks. Across Experiments 1 and 2, gaze toward consistently elicited larger N170 amplitudes than gaze away, independent of context, indicating robust early encoding of eye contact. In contrast, N2pc amplitudes were flexibly modulated: strongest when gaze was task-relevant, reduced when participants judged spatial direction. Cross-experiment analyses confirmed a graded decrease in gaze-related N2pc effects, while N170 remained stable. Finally, in Experiment 3, N170 and N2pc effects were absent when gaze was irrelevant. These results reveal a two-stage process in dynamic gaze perception: an early, context-independent perceptual encoding and a later, task-dependent attentional selection. The findings refine the Fast-Track Modulator Model by demonstrating that gaze processing is perceptually robust but attentionally flexible, indicating that gaze effects are conditional rather than automatic.

Significance statement Eye contact is a fundamental social signal, yet it remains debated whether its impact on perception and attention is automatic or shaped by context. Using EEG, we show that dynamic gaze processing unfolds in two distinct stages: an early perceptual encoding that is consistently enhanced for gaze shift toward an observer, and a later attentional selection that varies with task demands and resource availability. This two-stage profile challenges strictly automatic accounts and supports a flexible, context-dependent model of social attention. By demonstrating how communicative relevance gates the neural impact of eye contact, our findings refine current models of gaze perception and offer insights for understanding atypical social attention in conditions such as autism and social anxiety.

## Introduction

Eye gaze is a fundamental channel in human social interaction, signaling others’ focus of attention, intentions, and affective states, and shaping joint action and social inference from infancy to adulthood (Argyle and Cook 1976, Emery 2000, Farroni *et al.* 2002, Frischen *et al.* 2007). Unlike static laboratory images, genuine gaze in everyday life is dynamic. Shifts in gaze signal engagement or disengagement: a gaze shift toward suggests interaction readiness, while shifting away indicates withdrawal. Understanding these dynamic gaze cues provides crucial insights into social attention mechanisms.

The fast-track modulator (FTM) model (Senju and Johnson 2009) proposes that direct gaze is rapidly processed via subcortical pathways, granting it privileged and potentially obligatory access to attention. However, later studies question this strict automatic response. Behavioral evidence shows that looking directly at someone can increase alertness and social awareness (“watching eyes” effect; Conty *et al.* 2007, 2016, Hietanen *et al.* 2016, Myllyneva and Hietanen 2015) and can automatically draw attention (Friesen and Kingstone 1998). But its effect is not the same in every situation: the task, the meaning of the communication, and the amount of attention available can greatly change how gaze affects us (Hamilton 2016, Dalmaso *et al.* 2020).

Consistent with this flexible view, event-related potentials (ERPs) studies indicate that enhancements of N170 and N2pc to direct gaze emerge most reliably when eye contact is task-relevant, reflecting an interplay between fast-track activation and top-down control (Burra *et al.* 2019, Burra and Kerzel 2021, Tautvydaite and Burra 2024) and that this prioritization is shaped by social relevance. Thus, a key question is whether the FTM operates as a fixed, automatic detector of eye contact or as a flexible mechanism modulated by top-down goals.

ERPs allow us to trace the temporal cascade of gaze processing. The N170 indexes early face encoding (Bentin *et al.* 1996) and is reliably modulated by gaze direction (Schuller and Rossion 2001) and eye contact (for a review see Tautvydaitė *et al.* 2022). The N2pc, a contralateral posterior negativity emerging ∼200–300 ms, reflects spatial selection of salient or task-relevant stimuli (Luck and Hillyard 1994, Eimer 1996, Hickey *et al.* 2006, Luck and Kappenman 2011, Luck 2014). Together, these components allow us to capture a two-stage cascade of gaze processing: early perceptual encoding (N170) and later attentional selection (N2pc).

Dynamic gaze paradigms show enhanced neural responses to motion toward observers versus gaze aversion. Puce *et al.* (2000) showed early modulation of the N170 by gaze motion, with results varying by task. Conty *et al.* (2007) showed an early neural difference between gaze directed toward versus away from observers, indicating rapid prioritization of eye contact. Latinus *et al.* (2015) found N170 modulation occurs only during social judgments, suggesting gaze processing switches between spatial and social modes.

While prior work has mapped early perceptual correlates of eye contact, few studies have examined how attentional deployment unfolds across contexts. It remains unclear whether direct gaze automatically captures attention or whether attentional effects depend on the relevance of gaze. To probe the time course of attentional deployment to gaze contact, we measured both N170 and N2pc using contralateral measurements. The N170, also measured as a contralateral measure (Towler and Eimer 2015), provides a precise index of early perceptual encoding of eye contact. The N2pc, by contrast, is strictly contralateral and reflects the deployment of spatial attention to one hemifield. While N2pc effects are robust for emotionally or socially relevant faces (Wirth and Wentura 2023), it remains debated whether eye contact reliably elicits such attentional capture.

Building on this framework, the present study employed three EEG experiments designed to assess the role of task relevance in dynamic gaze processing while keeping visual stimulation and EEG procedures constant. Critically, Experiments 1 and 2 were designed for direct comparability, differing only in task instructions while using identical stimuli and analysis pipelines. In Experiment 1, participants judged whether gaze was directed toward or away from them, emphasizing communicative meaning (social task). In Experiment 2, they judged gaze direction (left vs. right), reducing its communicative relevance while maintaining attention to eye movements (non-social task). In contrast, Experiment 3 was designed as a control (boundary) condition, in which gaze signals were both task-irrelevant and unattended, while participants performed a central detection task.

This design allowed us to test a critical prediction: if gaze-related ERP components depend on task relevance, their amplitude should be reduced or absent when gaze cues are task-irrelevant and attention is directed elsewhere. Importantly, Experiment 3 was not intended to represent a third level along the same continuum as Experiments 1 and 2. Rather, it served to determine whether the neural effects observed under task-relevant conditions would persist when gaze signals were no longer behaviorally meaningful and could be reduced to low-level visual changes.

This manipulation tested three competing accounts of dynamic gaze processing. Our main prediction proposes two stages: the N170 indexes early perceptual encoding of eye contact, while the N2pc reflects later attentional selection shaped by task goals and resources. Under strict automaticity, both N170 and N2pc should show enhancements for gaze shift toward observers across all tasks, regardless of relevance. Under global context-sensitivity, both components should vary in parallel, being strongest for socially relevant eye contact, weaker for spatial direction judgments, and absent when gaze cues are task-irrelevant and attention is directed elsewhere. By contrasting these predictions across three matched experiments, this study tests whether gaze capture is automatic, partially flexible, or context-dependent.

## Experiment 1: Social task

### Method

#### Population

Twenty-two right-handed volunteers (12 female; mean age = 23.6 years, range 19–31) participated in Experiment 1. All met the inclusion criteria of being between 18 and 35 years old, with normal or corrected-to-normal vision and no history of neurological or psychiatric disorders. Participants were naïve to the purpose of the experiment and received course credit for their participation. The study was approved by the Ethics Committee of the University of Geneva, and informed consent was obtained from all participants.

The experiment was initially planned based on an a priori power analysis conducted with G*Power (Faul *et al.* 2007), targeting 80% power to detect a medium-sized within-subject effect (Cohen’s *d* = 0.45). This analysis indicated a required sample size of approximately 40 participants. Data collection included an interim evaluation at *N* = 22, motivated by consistency with prior ERP studies on gaze processing, which typically report sample sizes in the range of 20–25 participants (e.g. Conty *et al.* 2007; Latinus *et al.* 2015). Following this interim point, the observed effect size and data quality were evaluated, and data collection was not extended further. To further characterize the sensitivity of Experiment 1, a sensitivity analysis (G*Power; Faul *et al.* 2007) indicated that, for a paired-samples *t*-test with *N* = 22, α = 0.05, and 80% power, the minimal detectable effect size was approximately *d* = 0.62.

#### Stimuli and apparatus

High-resolution grayscale photographs of 10 male and 10 female faces with neutral expressions (¾ head deviation) were selected from Conty *et al.* (2007) and Latinus *et al.* (2015). Head orientation was counterbalanced across trials, such that each face was presented equally often with leftward and rightward head deviation. Face identities were fully counterbalanced across conditions to avoid low-level repetition effects. On each trial, two different identities were always presented simultaneously, ensuring that the same identity never appeared in both hemifields. Stimulus position was fully counterbalanced through mirroring, such that 50% of trials were presented in one spatial configuration and 50% in the mirrored configuration. This ensured that each identity and gaze condition appeared equally often in the left and right visual fields, preventing potential confounds related to spatial asymmetries or socially meaningful gaze configurations (e.g. gaze directed toward another face).

Images were standardized for luminance and contrast, using SHINE Toolbox (Willenbockel *et al.* 2010) and subtended at a visual angle of 6° × 8°. Two faces were presented per trial, one to the left and one to the right of fixation (eccentricity ≈ 4°). Each face initially displayed an averted gaze (15°). After 500 ms, the gaze of one face shifted over 200 ms to become either direct (0°) or further averted (30°), while the other face closed its eyes. These dynamic gaze changes were implemented using apparent motion (i.e. successive static images presented in close temporal succession), following Conty *et al.* (2007) and Latinus *et al.* (2015). Two different identities were always displayed.

Stimuli were presented on a 24-inch LCD monitor with a refresh rate of 60 Hz and resolution of 1920 × 1080 pixels. Participants were seated approximately 70 cm from the screen, and responses were collected using a standard keyboard. Stimulus presentation and timing were controlled using MATLAB with Psychtoolbox extensions (Brainard 1997, Kleiner *et al.* 2007).

#### Procedure

**Figure 1:**
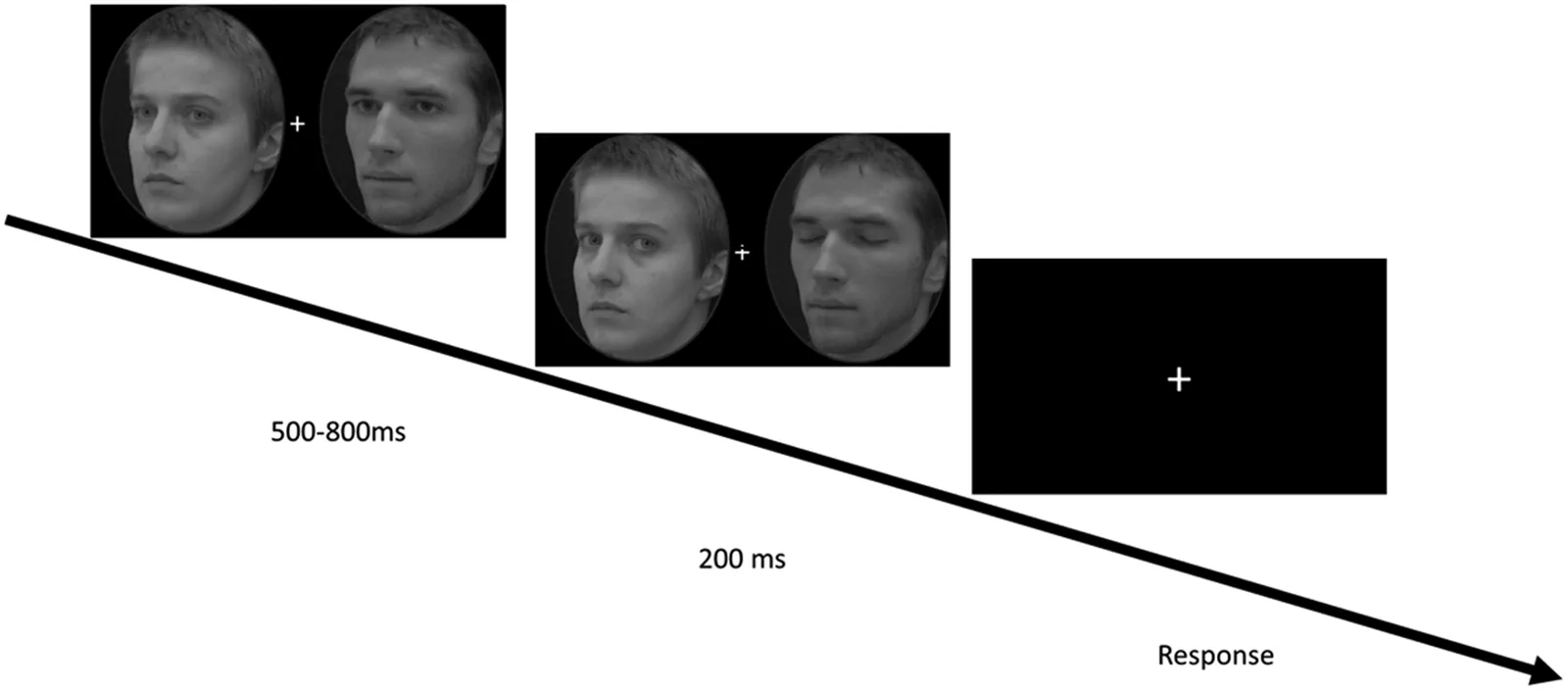
Experimental procedure. Schematic representation of the trial sequence. A central fixation cross was presented (500–1500 ms), followed by two faces displayed bilaterally (4° eccentricity). Both faces initially showed an averted gaze (15°; 500 ms). The gaze of one face then shifted dynamically over 200 ms to either direct gaze (0°; toward) or further averted gaze (30°; away), while the competing face closed its eyes. Participants indicated whether the target face’s gaze endpoint was directed toward or away from them. Trials were separated by a variable inter-trial interval (800–1200 ms).

As depicted in Fig. 1, each trial began with a central fixation cross (500–1500 ms). Participants were instructed to maintain fixation and avoid eye movements. Two faces then appeared, one to the left and one to the right of fixation (4°), with both pairs of eyes initially averted by 15° (500 ms). Next, the gaze of one face shifted dynamically (200 ms) to either establish a direct gaze (0°= Toward) or become further averted (30° = Away), while the competing face closed its eyes.

Participants pressed one of two buttons to indicate whether the target face’s gaze endpoint was directed toward or away from them, with instructions emphasizing both speed and accuracy. Trials were separated by a variable inter-trial interval (800–1200 ms). Each participant completed 240 trials, divided into four blocks of 60 trials, with equal numbers of gaze toward and gaze away. Face identity and gender were randomized across trials, and the same identity was never presented simultaneously in both hemifields.

The fixation cross included a slight luminance change in a single pixel, equally placed either above or below the intersection (50% each). Although this pixel manipulation was later used as the target in Experiment 3, it remained entirely task-irrelevant in Experiments 1 and 2. Its inclusion therefore ensured strict visual continuity across experiments without introducing functional overlap in task demands. This distinction is critical, as Experiments 1 and 2 were designed to be directly comparable in terms of task relevance, whereas Experiment 3 specifically manipulated the behavioral relevance of gaze signals.

#### Electrophysiological recording and preprocessing

Electroencephalography (EEG) data were recorded using a 64-channel BioSemi ActiveTwo system, with electrodes positioned according to the international 10-20 system. Horizontal and vertical electrooculograms (EOGs) were recorded to monitor eye movements and blinks. EEG signals were sampled at 1024 Hz, downsampled to 256 Hz, and filtered offline with a bandpass filter ranging from 0.1 to 30 Hz. Ocular artifacts were removed using independent component analysis (ICA), and data were re-referenced to the average of all electrodes (see Tautvydaite and Burra 2024, for the same procedure). Epochs extended from −100 to 350 ms relative to gaze-shift onset and were baseline-corrected using a −100 to 0 ms interval. Trials were rejected if they contained blinks (VEOG ± 60 μV), saccades (HEOG ± 30 μV), excluded to prevent contamination of lateralized ERP components such as the N2pc, or voltage fluctuations exceeding ± 100 μV at any electrode. On average, 82% of trials were retained per participant, leaving a mean of 210 valid trials per condition. Rejection criteria were applied consistently across all experiments.

#### Behavioral data analysis

Accuracy (ACC) and reaction times (RTs) were measured for each experiment. Repeated-measures analyses of variance (ANOVAs) were conducted to assess the effects of Gaze Direction (Toward vs. Away) on ACC and RTs.

#### ERP data analysis

**Figure 2:**
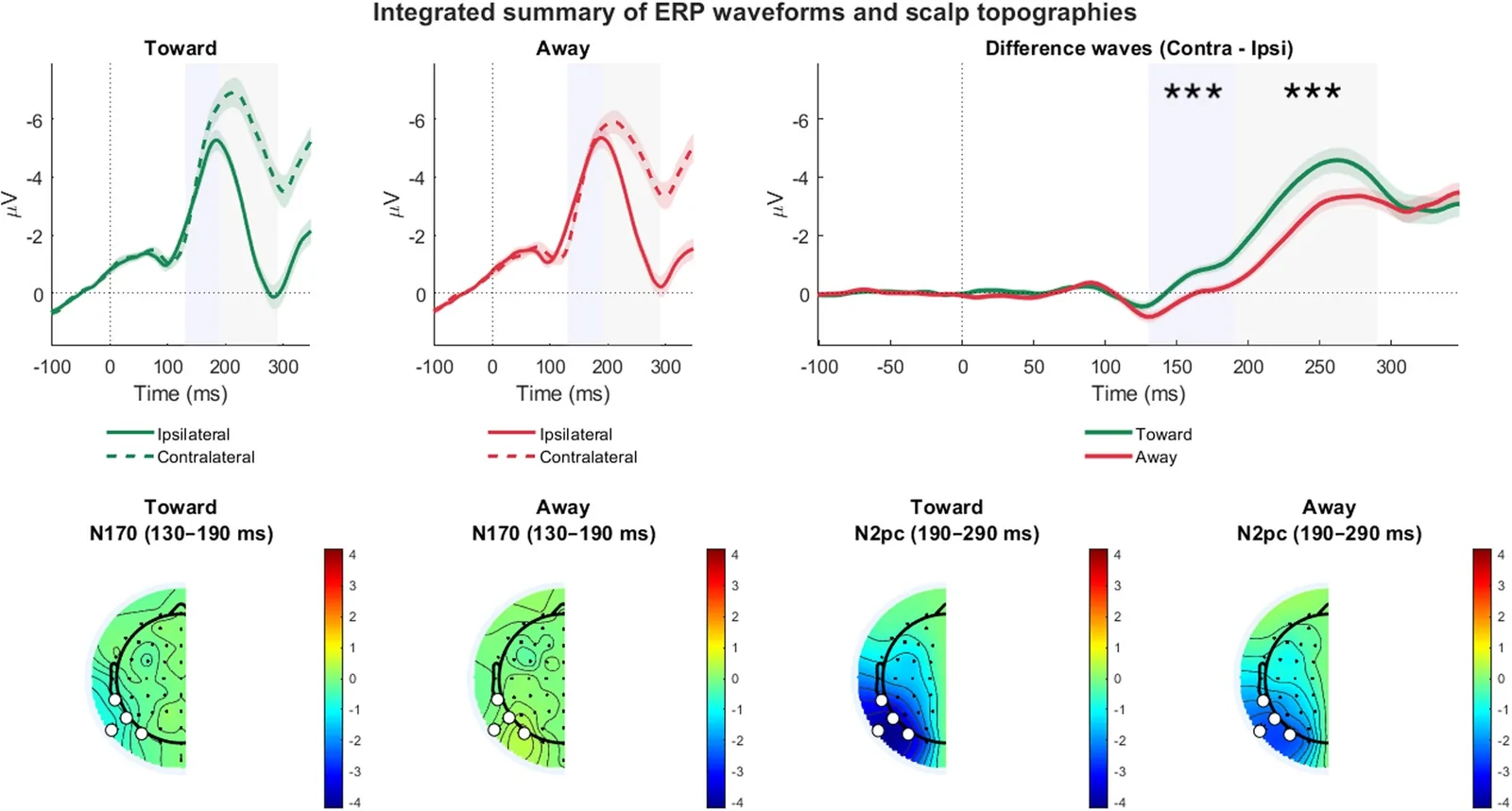
Grand-average ERPs at posterior sites for Experiment 1. Figure shows on top, the contralateral and ipsilateral ERPs at posterior electrodes (PO7/PO8, P7/P8, P9/P10, TP9/TP10) for Toward and Away conditions, with corresponding difference waves (contra–ipsi), shown to index lateralized processing. Shaded areas represent the time windows used to quantify the N170 (130–190 ms, dark gray) and the N2pc (190–290 ms, light gray). On bottom, the scalp topographies for the N170 and N2pc time windows. White dots denote ROI electrodes; color scale in µV. Grand-average event-related potentials (ERPs) recorded at posterior electrodes as a function of gaze direction and task relevance. Negative is plotted upward.

ERP waveforms were time-locked to the onset of the gaze shift and averaged separately for each condition and participant. The mean amplitudes of the N170 and N2pc components were measured at a large cluster of posterior electrodes (PO7/PO8, P7/P8, P9/P10, TP9/TP10) as the difference between the contralateral and ipsilateral sites relative to the target stimulus, within the 130–190 ms and 190–290 ms time windows (as in Towler *et al.* 2015), respectively, as depicted in Fig. 2. Repeated-measures ANOVAs were conducted with factors including Gaze Shift (Toward vs. Away) and Electrode Site (PO7/PO8, P7/P8, P9/P10, TP9/TP10). Greenhouse-Geisser corrections were applied when necessary, and effect sizes were reported using partial eta squared (ηp^2^). The primary dependent measure was the contralateral–ipsilateral difference, which indexes lateralized neural activity. Statistical comparisons against zero were applied to the difference wave itself, reflecting the presence of reliable contra-ipsilateral processing rather than absolute deviations from baseline. Complementary Bayesian analyses were conducted to quantify the strength of evidence for the N2pc observed effects.

The full dataset (Experiments 1–3) supporting this study is available at the Open Science Framework (OSF): https://osf.io/vbrju/.

### Results

#### Behavioral results

Accuracy was higher for away than toward gaze, *t*(21) = 2.28, *P* = .033, *d *= 0.48. Accuracy was higher for gaze away (*M *= 0.93, *SD *= 0.07) than for gaze toward (*M *= 0.90, *SD *= 0.07). In contrast, response times did not differ between conditions (*P* = .75).

#### ERP results

##### N170

The ANOVA yielded a significant main effect of Gaze, *F*(1, 21) = 16.29, *P* < .001, ηp^2^ = 0.43. N170 amplitudes were significantly larger for toward gaze shifts (*M *=* −*0.52 µV, *SE *= 0.13) than for away gaze shifts (*M *= 0.18 µV, *SE *= 0.14). No Gaze × Electrode interaction emerged (*F *< 1). Using one-sample *t*-tests against zero, the toward gaze condition elicited a reliable N170 negativity compared to baseline, *t*(21) = −3.79, *P* < .001, *d *= 0.80, whereas the away condition did not (*P* = .23).

##### N2pc

The ANOVA yielded a significant main effect of Gaze, *F*(1, 21) = 27.53, *P* < .001, ηp^2^ = 0.57. N2pc amplitudes were significantly larger for toward gaze shifts (*M *=* −*3.80 µV, *SE *= 0.38) than for away gaze shifts (*M *=* −*2.47 µV, *SE *= 0.26). A Bayesian paired-samples *t*-test provided extreme evidence for the effect (BF_10_ = 1076). No Gaze × Electrode interaction emerged (*F *< 1). Using one-sample *t*-tests against zero, both the toward and away gaze conditions elicited reliable N2pc negativities compared to baseline (all *ts* > *−*9.9, all *ps* < .001, all *ds* > 2.12).

### Discussion: Experiment 1

Experiment 1 tested whether dynamic gaze endpoints capture attention during socially relevant eye contact. ERP measures showed stronger responses to gaze toward than gaze away. N170 amplitudes were enhanced for gaze toward, indicating enhanced face encoding (Bentin *et al.* 1996, Tautvydaitė *et al.* 2022). Additionally, gaze toward elicited a larger N2pc, showing that socially meaningful gaze shifts engage attentional selection. These results align with the Fast-Track Modulator Model, where gaze cues exert maximal influence with social significance (Senju and Johnson 2009, Burra *et al.* 2019). The findings extend ERP work on neural differences between gaze motions (Conty *et al.* 2007) and dynamic gaze processing (Latinus *et al.* 2015), suggesting eye contact enhances perceptual encoding at ∼170 ms, followed by spatial attention allocation at ∼200–300 ms. Behaviorally, participants showed higher accuracy for averted gaze, likely due to its less ambiguous directional meaning compared to direct gaze’s self-referential connotations (Hamilton 2016; Hietanen *et al.* 2016). This shows neural prioritization of eye contact doesn’t necessarily improve task performance (Riechelmann *et al.* 2021). Experiment 1 shows that with emphasized social meaning, gaze toward enhances perceptual encoding and attentional allocation, supporting top-down modulation in gaze perception and setting up Experiment 2's examination of these effects during non-social tasks.

## Experiment 2: Non-social

Experiment 2 examined whether dynamic gaze shifts capture attention when eye contact is not socially relevant. Participants judged gaze movement direction (left vs. right) rather than gaze contact establishment. This maintained attention to eye motion while reducing its social value. According to the Fast-Track Modulator Model (Burra *et al.* 2019), removing explicit eye contact references should weaken but not eliminate its influence on attention. We predicted N170 amplitudes would remain larger for gaze toward than away, while N2pc amplitudes would still differentiate between directions but with reduced magnitude versus Experiment 1.

### Method

The methods were identical to those of Experiment 1, except that participants were required to detect the direction of the gaze shift (left vs. right), i.e. a non-social judgment. Twenty-two additional healthy adults (11 female; age range = 18–30) participated in the study. Participants indicated the direction of the gaze shift by pressing one of two buttons. All other stimulus parameters, EEG recordings, and ERP analyses were identical to those used in Experiment 1. An a priori power analysis based on Experiment 1 was conducted using G*Power (Faul *et al.* 2007) for a paired-samples *t*-test with α = 0.05 and power = 0.80. This analysis indicated that a minimum of nine participants would be sufficient to detect the large effect size observed in Experiment 1 (*dz *= 1.16). We retained a sample size of 22 participants to ensure direct comparability across experiments and consistency with prior literature. On average, 83% of trials were retained per participant, leaving a mean of 213 valid trials per condition.

### Results

#### Behavioral results

Accuracy was higher for away than toward gaze shifts, *t*(21) = 5.8, *P* < .001, *d *= 1.23. Participants were more accurate for away gaze shifts (*M *= 0.97, *SD *= 0.02) than for toward gaze shifts (*M *= 0.94, *SD *= 0.02). Reaction times did not differ significantly between conditions (*P* = .77).

#### ERP results

##### N170

**Figure 3:**
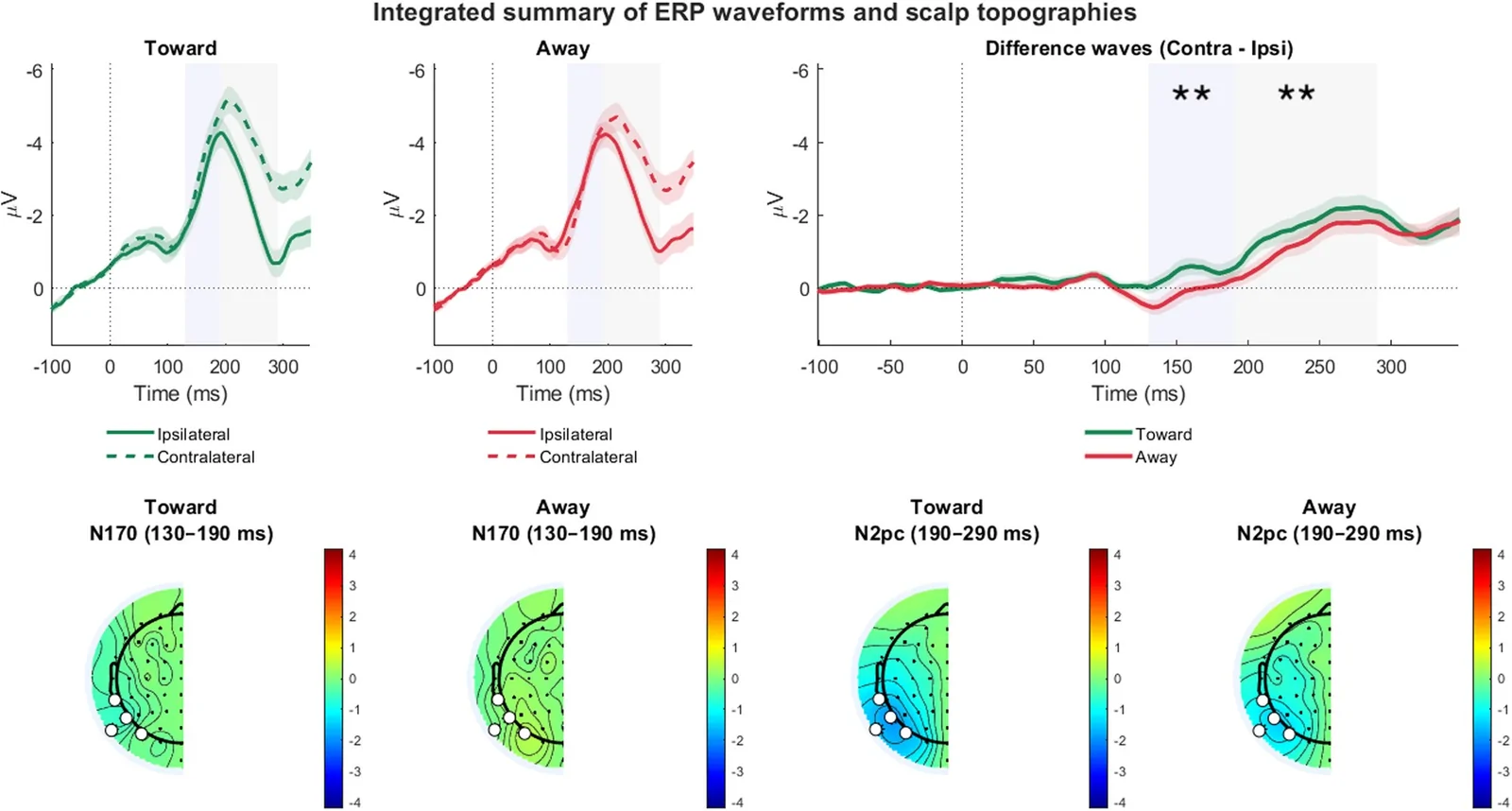
Grand-average ERPs at posterior sites for Experiment 2. Figure shows on top, the contralateral and ipsilateral ERPs at posterior electrodes (PO7/PO8, P7/P8, P9/P10, TP9/TP10) for Toward and Away conditions, with corresponding difference waves (contra–ipsi), shown to index lateralized processing. Shaded areas represent the time windows used to quantify the N170 (130–190 ms, dark gray) and the N2pc (190–290 ms, light gray). On bottom, the scalp topographies for the N170 and N2pc time windows. White dots denote ROI electrodes; color scale in µV. Grand-average event-related potentials (ERPs) recorded at posterior electrodes as a function of gaze direction and task relevance. Negative is plotted upward.

The ANOVA yielded a significant main effect of Gaze, *F*(1, 21) = 12.37, *P* < .002, ηp^2^ = 0.27. N170 amplitudes were significantly larger for toward gaze shifts (*M *=* −*0.39 µV, *SE *= 0.19) than for away gaze shifts (*M *= 0.13 µV, *SE *= 0.20; see Fig. 3). No Gaze × Electrode interaction was observed (*F *< 1). However, one-sample *t*-tests against zero, at the Experiment 2 level, neither the toward nor the away gaze condition elicited a reliable N170 negativity relative to baseline (toward: *t*(21) = *−*1.98, *P* = .061, *d *=* −*0.42; Away: *t*(21) = 0.67, *P* = .51, *d *= 0.14).

##### N2pc

The ANOVA yielded a significant main effect of Gaze, *F*(1, 21) = 8.51, *P* < .008, ηp^2^ = 0.29. N2pc amplitudes were significantly larger for toward gaze shifts (*M *=* −*1.68 µV, *SE *= 0.27) than for away gaze shifts (*M *=* −*1.22 µV, *SE *= 0.24; see Fig. 3). A Bayesian analysis indicated anecdotal-to-moderate evidence for the effect (BF_10_ = 2.55). No Gaze × Electrode interaction was observed (*F *< 2.5). Using one-sample *t*-tests against zero, both gaze conditions elicited reliable N2pc negativities compared to baseline (all *ts *=* −*5.07, all *ps* < .001, all *ds* > 1.08).

### Cross-experiment ERPs comparison (experiment 1 vs. experiment 2)

#### Behavioural results

Behavioral data were analyzed using a mixed-design ANOVA with Gaze (toward vs. away) as a within-subject factor and Experiment (1 vs. 2) as a between-subject factor.

For reaction times, no main effect of Gaze was observed, *F*(1, 42) = 0.16, *P* = .687, ηp^2^ = .004, nor a Gaze × Experiment interaction, *F*(1, 42) = 0.02, *P* = .865, ηp^2^ < 0.001. However, a significant main effect of Experiment was found, *F*(1, 42) = 23.9, *P* < .001, ηp^2^ = 0.363, indicating overall differences in response times between tasks with longer RT for Experiment 1 than Experiment 2.

For accuracy, a significant main effect of Gaze was observed, *F*(1, 42) = 21.71, *P* < .001, ηp^2^ = 0.346, reflecting higher accuracy for away compared to toward gaze. The Gaze × Experiment interaction was not significant, *F*(1, 42) = 0.36, *P* = .55, ηp^2^ = 0.008, indicating that this effect was consistent across experiments. A main effect of Experiment was also found, *F*(1, 42) = 4.64, *P* = .037, ηp^2^ = 0.1, suggesting overall differences in task difficulty, with a higher ACC for Experiment 2 than Experiment 1.

#### Lateralized ERPs results

To compare task effects, mixed-design ANOVAs were conducted with Gaze as a within-subject factor and Experiment (1 vs. 2) as a between-subject factor. These analyses focused on Experiments 1 and 2, which were matched in design and sample size, allowing a direct comparison of task relevance.

##### N170

The repeated-measures ANOVA revealed a significant main effect of Gaze, *F*(1, 42) = 28.67, *P* < .001, ηp^2^ = 0.41, with stronger N170 negativities for toward gaze (*M *=* −*0.46 µV, *SE *= 0.12) than for away gaze (*M *= 0.16 µV, *SE *= 0.12). A significant main effect of electrode was also observed, *F*(3, 126) = 8.12, *P* < .001, ηp^2^ = 0.16. No other effects reached significance, including Gaze × Experiment (*F*(1, 42) = 0.60, *P* = .444, ηp^2^ = 0.01), Gaze × Electrode (*F*(3, 126) = 0.63, *P* = .599, ηp^2^ = 0.02), and Gaze × Electrode × Experiment (*F*(3, 126) = 0.60, *P* = .615, ηp^2^ = 0.01). The between-subjects effect of Experiment was also nonsignificant, *F*(1, 42) = 0.04, *P* = .837, ηp^2^ < 0.01.

To assess the overall presence of lateralized activity associated with gaze direction across Experiments 1 and 2, one-sample *t*-tests against zero showed that N170 amplitudes for toward gaze were significantly different from zero, *t*(43) = *−*3.82, *P* < .001, *d *=* −*0.58, whereas away gaze amplitudes did not differ significantly from zero, *t*(43) = 1.30, *P* = .202, *d *= 0.20.

##### N2pc

**Figure 4:**
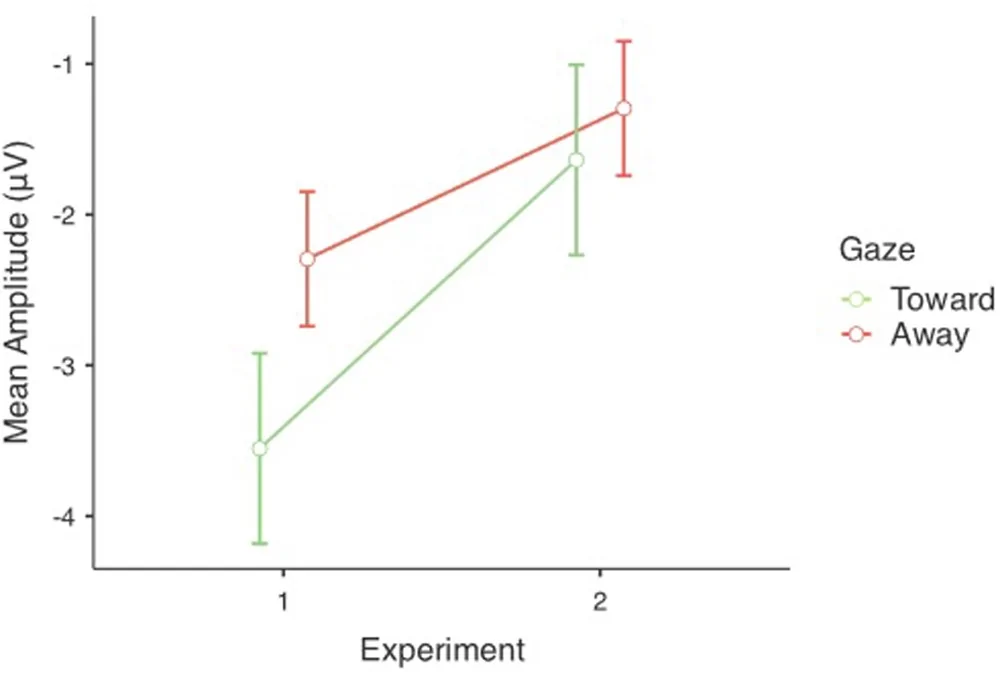
N2pc component across experiments. Interaction between gaze direction (toward vs. away) and experiment on mean ERP amplitudes. Negative values are plotted upward. Error bars represent 95% confidence intervals.

To directly compare the impact of task relevance, we conducted a repeated-measures ANOVA with Gaze (toward vs. away) as a within-subjects factor and Experiment (1 vs. 2) as a between-subjects factor. The analysis revealed a robust main effect of Gaze, *F*(1,42) = 37.01, *P* < .001, ηp^2^ = 0.46, indicating larger N2pc amplitudes for gaze shifts directed toward the observer (*M *=* −*2.78 µV, *SE *= 0.23) than for gaze shifts directed away (*M *=* −*1.89 µV, *SE *= 0.18). Importantly, the Gaze × Experiment interaction was significant, *F*(1,42) = 9.23, *P* = .004, ηp^2^ = 0.18, demonstrating that the size of the gaze effect differed across experiments (see Fig. 4). A significant main effect of Experiment, *F*(1,42) = 16.5, *P* < .001, ηp^2^ = 0.28, further indicated that overall N2pc amplitudes were larger in Experiment 1 (*M *=* −*3.15 µV, *SE *= 0.28) than in Experiment 2 (*M *=* −*1.50 µV, *SE *= 0.22). No significant effects involving electrodes were observed (*ps* > .20).

To further characterize the Gaze × Experiment interaction, simple main effects of Gaze were tested separately within each experiment (see Main effects of Gaze in Experiments 1 and 2 results sections), confirming a significant effect in both tasks but markedly stronger in Experiment 1 than in Experiment 2.

To further quantify this difference, the magnitude of the gaze effect (toward−away) was compared between experiments, using an independent-*t* test. This analysis revealed that the gaze effect was significantly greater in Experiment 1 than in Experiment 2, *t*(42) = *−*2.82, *P* = .007, *d *=* −*0.84, consistent with the interaction observed in the ANOVA. A Bayesian comparison confirmed strong evidence for a difference in effect size between Experiment 1 and Experiment 2 (BF_10_ = 22.2).

### Discussion: Experiment 2

Experiment 2 examined dynamic gaze processing under a non-social task, in contrast to the social task used in Experiment 1. Although both tasks elicited N2pc responses, the gaze direction setup was less social here than in Experiment 1. This suggests attentional prioritization by gaze toward the observer depends on its social value. N170 amplitudes remained larger for gaze toward than away, replicating Experiment 1 and showing eye contact enhances early face encoding regardless of task. However, in Experiment 2, although a significant contra-ipsilateral difference was observed, neither condition showed a statistical difference from baseline. This suggests that the effect reflects a relative lateralized modulation rather than a robust absolute N170 response as observed in Experiment 1. This pattern may also reflect reduced or less precisely time-locked activity, which reduces its detectability within the predefined analysis window. The N2pc showed reduced differentiation between gaze directions when participants focused on spatial direction rather than social intent. Behaviorally, participants were more accurate for gaze away than toward, likely reflecting clearer directional information in averted gaze. These findings extend the Fast-Track Modulator Model (Senju and Johnson 2009, Burra *et al.* 2019). They show gaze processing is shaped by perceptual encoding and task goals: Early neural responses to gaze toward were robust, whereas attentional selection depended on task demands. They highlight how contextual framing affects gaze research outcomes depending on social or spatial emphasis. Importantly, behavioral results did not mirror the electrophysiological findings. While gaze toward the observer enhanced neural responses (N170 and N2pc), participants were more accurate when judging away gaze, and reaction times did not differ between conditions. This suggests that early perceptual and attentional prioritization of eye contact does not necessarily translate into improved behavioral performance, particularly when task demands rely on spatial discrimination. Building on this, Experiment 3 tested whether dynamic gaze affects attention when it is fully engaged by a demanding central task.

## Experiment 3: Task-irrelevant

Experiment 3 was designed as a control condition to assess whether the neural effects observed in Experiments 1 and 2 depend on the behavioral relevance of gaze cues. In contrast to the previous experiments, participants were instructed to perform a central detection task, while gaze direction was entirely irrelevant to task performance. This design allowed us to test whether gaze-related ERP components, such as the N170 and N2pc, would still be elicited when attention was directed away from gaze signals. If these components depend on task relevance, their amplitude should be reduced or absent under these conditions. Importantly, the physical properties of the stimuli, including the dynamic gaze shifts and associated visual changes, were identical to those used in Experiments 1 and 2. This allowed us to assess whether the effects observed previously could be explained by low-level visual features. If gaze-related effects were driven solely by such low-level factors (e.g. motion or eye aperture changes), they should persist even when gaze cues are task-irrelevant. Accordingly, if gaze processing depends on behavioral relevance rather than purely sensory factors, we expected a marked reduction or absence of N170 and N2pc modulations in this condition.

### Method

The general procedures were identical to those used in Experiment 1 and 2, except as noted. Experiment 3 was designed as a control condition in which gaze cues were task-irrelevant. Accordingly, a reduced sample size was used based on power estimates derived from Experiment 1. Ten participants (six females; age range = 18–28, *M *= 22) took part in the study. Each trial followed the same sequence as in Experiments 1 and 2: a central fixation cross was presented, followed by two peripheral faces that performed dynamic gaze shifts. The only change concerned the central task. Participants were instructed to perform a detection task at central location. Specifically, immediately at the gaze shift, the fixation cross contained a missing pixel in either its upper or lower segment (50% of trials each). Participants pressed a designated key to indicate the location of the missing pixel, with instructions emphasizing both speed and accuracy. On average, 89% of trials were retained per participant, leaving a mean of 228 valid trials per condition.

### Results

#### Behavioral results

Performance on the central task was consistently high (above 0.90) and unaffected by peripheral gaze cues. Accuracy did not differ significantly between gaze conditions, *t*(9) = 0.92, *P* = .38 (*M *= 0.97 vs. *M *= 0.98). Reaction times also showed no reliable effect of gaze direction, *t*(9) = 1.30, *P* = .22 (*M *= 379 ms vs. *M *= 377 ms).

#### ERP results

##### N170

**Figure 5:**
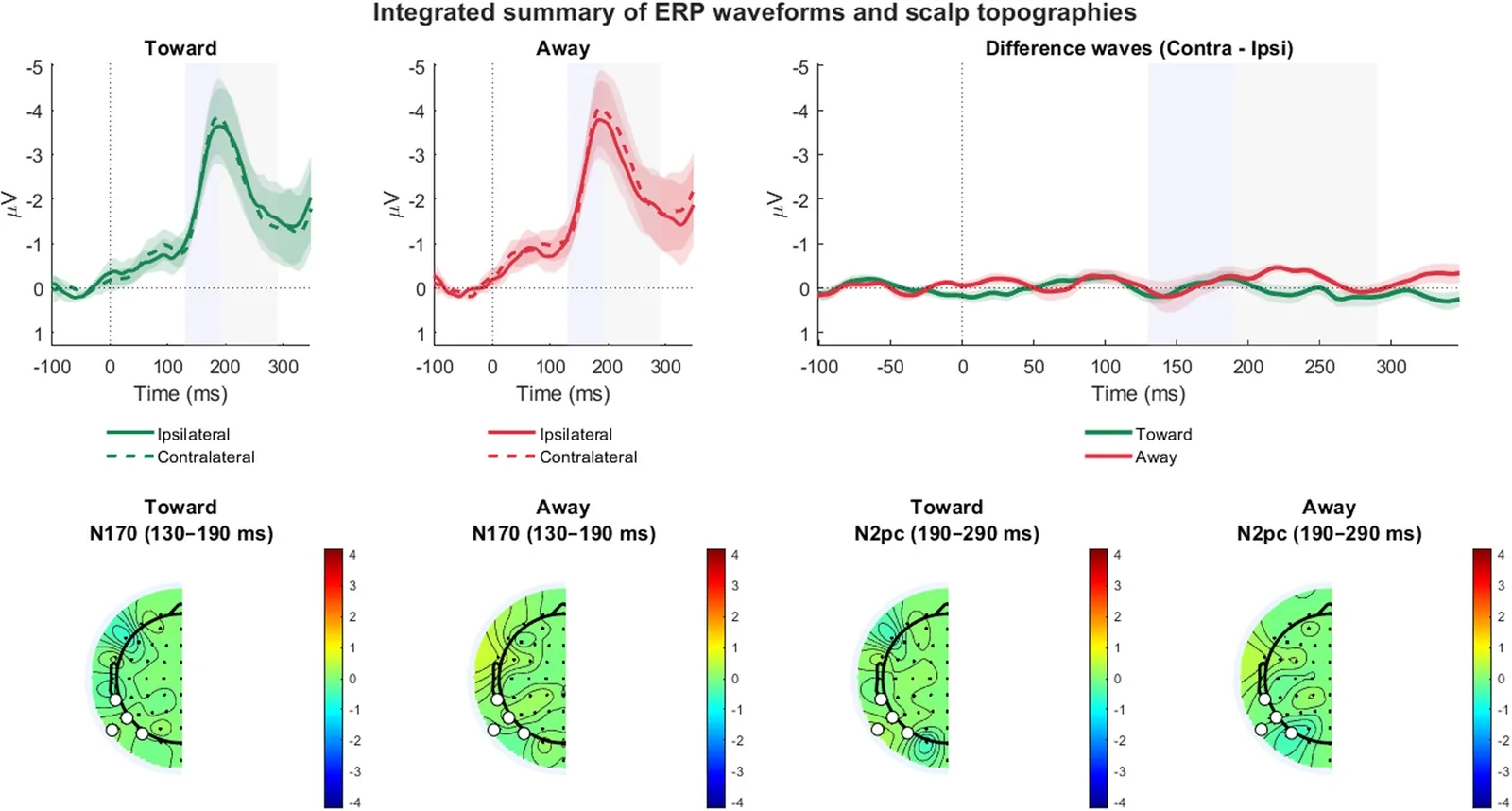
Grand-average ERPs at posterior sites for experiment 3. Figure shows on top, the contralateral and ipsilateral ERPs at posterior electrodes (PO7/PO8, P7/P8, P9/P10, TP9/TP10) for toward and away conditions, with corresponding difference waves (contra–ipsi), shown to index lateralized processing. Shaded areas represent the time windows used to quantify the N170 (130–190 ms, dark gray) and the N2pc (190–290 ms, light gray). On bottom, the scalp topographies for the N170 and N2pc time windows. White dots denote ROI electrodes; color scale in µV. Grand-average event-related potentials (ERPs) recorded at posterior electrodes as a function of gaze direction and task relevance. Negative is plotted upward.

As depicted in Fig. 5, the repeated-measures ANOVA revealed no significant effects of Gaze, *F*(1, 9) = 0.01, *P* = .971, or Electrode, *F*(3, 27) = 0.61, *P* = .616, and no reliable Gaze × Electrode interaction, *F*(3, 27) = 1.64, *P* = .203. Using one-sample *t*-tests against zero, neither the toward nor the away gaze condition elicited significant deviations from baseline (Toward: *t*(9) = 0.27, *P* = .795; Away: *t*(9) = *−*0.16, *P* = .877).

##### N2pc

N2pc amplitudes did not differ significantly between toward gaze (*M *=* −*0.05 μV, *SE *= 0.45) and away gaze (*M *=* −*0.21 μV, *SE *= 0.34), *F*(1, 9) = 3.85, *P *= .08. Bayesian analyses indicated inconclusive evidence (BF_10_ = 1.23; BF_01_ = 0.85). To assess whether a lateralized attentional response was present irrespective of gaze direction, a one-sample *t*-test against zero was conducted on N2pc amplitudes collapsed across conditions (toward and away). This analysis revealed no reliable N2pc, *t*(9) = 0.62, *P* = .54, *d *= 0.21. A complementary Bayesian one-sample *t*-test provided anecdotal evidence supporting the absence of a reliable effect (BF_01_ = 2.27) (Fig. 5).

### Discussion: Experiment 3

Experiment 3 served as a control condition in which gaze cues were task-irrelevant and attention was directed toward a central task. Under these conditions, gaze-related ERP effects were markedly reduced, supporting the idea that these neural responses depend on the behavioral relevance of gaze cues. Importantly, this pattern also argues against an interpretation in terms of low-level visual confounds. Because the physical stimulation, including apparent motion and eye-related changes, was identical across experiments, any effect driven purely by low-level visual features would be expected to persist in Experiment 3. The absence (or likely trend inversion) of such effects therefore suggests that the modulations observed in Experiments 1 and 2 cannot be explained solely by low-level visual properties. However, because attention was directed centrally, the present design does not allow us to fully differentiate the effects of task irrelevance from those of spatial attentional allocation. The reduction of gaze-related effects may therefore reflect both the lack of behavioral relevance and the prioritization of centrally presented information over peripheral stimuli. Future studies using designs in which competing tasks are presented in the periphery or combining EEG with independent measures of attentional allocation, would allow a more precise separation of these factors.

## General discussion

The present study examined how dynamic gaze endpoints influence both early perceptual encoding and later attentional selection. Using the N170 and N2pc as electrophysiological markers, we manipulated the social relevance of gaze and the availability of attentional resources across three experiments. Three main results emerged. First, when eye contact was explicitly socially relevant (Experiment 1), gaze directed toward an observer enhanced both N170 and N2pc amplitudes, indicating robust encoding and prioritization of communicative signals. Second, when participants judged gaze direction, i.e. non-socially relevant (Experiment 2), N170 remained sensitive to gaze toward the observer, but the N2pc effect was attenuated, demonstrating reduced attentional prioritization. Third, when gaze cues were task-irrelevant and attention was directed toward a demanding central task (Experiment 3), neither N170 nor N2pc showed reliable modulation, suggesting that dynamic gaze cues fail to capture attention when gaze cues are task-irrelevant and attention is directed elsewhere. Cross-experiment analyses confirmed that N170 effects were consistent across Experiment 1 and 2, while N2pc effects decreased progressively from Experiment 1 to 2. Bayesian analyses converged with frequentist results, supporting a graded reduction of the effect across experiments.

Importantly, Experiment 3, designed as a control condition in which gaze cues were task-irrelevant, argues against an interpretation in terms of low-level visual confounds. Because the physical stimulation was identical across experiments, including dynamic gaze motion and eye-related changes, any effect driven solely by low-level visual properties would be expected to persist across conditions. The marked reduction of effects in Experiment 3 therefore indicates that the observed modulations cannot be explained solely by sensory factors. As such, it was not included in the same cross-experiment model as Experiments 1 and 2. Future studies using fully matched designs across all conditions would be valuable to extend this comparison.

These findings refine current models of social attention. Conty *et al.* (2007) showed that direct and averted gaze motions separate within 160–210 ms, engaging a social brain network. Our results extend this by demonstrating a comparable lack of convergence at later attentional stages: direct gaze enhances attentional selection (N2pc), but only when its communicative value is emphasized. Latinus *et al.* (2015) further reported that N170 responses to gaze motion vary with task framing. While their mixed-task design yielded context effects at the N170 stage, our between-experiment design revealed a clearer division of labor: N170 reflected stable, context-independent encoding of gaze shift, whereas N2pc was flexibly modulated by task demands.

The data challenge the notion that gaze automatically captures attention. The original Fast-Track Modulator Model (Senju and Johnson 2009) proposed that direct gaze triggers a subcortical route, conferring obligatory attentional priority. Our results instead support updated formulations (Senju and Johnson 2009; Burra *et al.* 2019), in which gaze-driven effects operate along a continuum: amplified when communicative meaning is high, attenuated when it is reduced, and absent when resources are diverted elsewhere. This graded view highlights the central role of top-down factors in shaping gaze effects.

A limitation of the present study is the lack of convergence between neural and behavioral measures. Reaction times did not differ between conditions, likely because both involved comparable perceptual and decisional demands. In contrast, gaze directed toward the observer reliably enhanced ERP markers, indicating prioritized processing at early perceptual (N170) and attentional (N2pc) stages. Behaviorally, however, participants were more accurate when judging averted gaze endpoints, likely because averted gaze provides clearer directional information. This pattern suggests that while direct gaze benefits early perceptual encoding and attentional selection, these neural advantages do not necessarily translate into faster or more accurate overt responses when task demands rely on spatial discrimination. This interpretation is further supported by exploratory brain–behavior analyses (see Supplementary Materials), which revealed no reliable correlations between gaze-related ERP amplitudes (N170, N2pc) and behavioral measures (accuracy, reaction times). This absence of association reinforces the conclusion that neural prioritization of gaze cues does not directly translate into overt behavioral performance. More generally, the N2pc modulation reflects differences in attentional allocation that do not obligatorily lead to measurable behavioral facilitation. This discrepancy highlights that neural prioritization of socially relevant cues, such as eye contact, operates at processing stages that may remain partially independent from response-level performance.

Notably, the behavioral difference in task difficulty between experiments, similar to Latinus *et al.* (2015), does not straightforwardly account for the ERP pattern observed: whereas the N2pc was reduced in Experiment 2, the lateralized N170 remained relatively stable across experiments, suggesting that the two components were differentially sensitive to the task manipulation rather than uniformly affected by general performance differences. The precise mechanisms underlying this selective modulation, whether driven by task-specific attentional demands, differences in communicative relevance, or other contextual factors, remain an open question for future research.

Additionally, because attention was directed toward a central task in Experiment 3, the present design does not allow us to fully disentangle the effects of task irrelevance from those of spatial attentional allocation. Future studies using fully matched designs or independent measures of attentional engagement would help clarify this distinction.

Finally, the present findings are based on apparent motion rather than continuous physical motion. Although apparent motion produces a compelling percept of gaze change, it differs from true motion in that it is generated from successive static images rather than continuous spatiotemporal input. These two forms of motion may rely on partially distinct neural mechanisms, with apparent motion engaging higher-level perceptual interpolation processes, whereas continuous motion may more directly recruit motion-sensitive visual pathways. As a result, the present findings may primarily reflect the processing of perceived gaze change rather than physically continuous motion per se. Future studies using continuous motion stimuli (e.g. videos or animations) would be necessary to determine the extent to which the observed effects generalize to more naturalistic gaze dynamics.

Our findings converge with a growing literature showing that gaze effects depend on task goals, social meaning, and context. Hamilton (2016) emphasized the role of communicative relevance in gaze cueing. Dalmaso *et al.* (2020) reviewed evidence that gaze effects vary with emotion, social status, and group membership. At the neural level, Breil *et al.* (2022) found that direct gaze captures attention only in socially meaningful contexts, and Wirth and Wentura (2023) showed that N2pc to emotional expressions depends on task framing. Recently, Chen *et al.* (2025) observed stronger N2pc for averted than direct gaze, Binetti *et al.* (2017) demonstrated that gaze shifts away from the observer can sometimes be prioritized in temporal order judgments, and Riechelmann *et al.* (2021) reported an averted-gaze advantage in a gaze discrimination task under brief presentation conditions. Together, these findings reinforce the conclusion that gaze effects are not reflexive but flexibly modulated.

A further methodological consideration concerns our use of dynamic gaze stimuli. Dynamic displays offer higher ecological validity for social interaction but may engage additional motion-sensitive processes. Some discrepancies across studies could stem from these differences: static gaze stimuli elicit weaker ERP effects while allowing better control of visual factors (Tautvydaitė *et al.* 2022). Future work comparing static and dynamic gaze under identical conditions could clarify if N170 and N2pc responses generalize beyond motion-based paradigms.

Finally, our results may inform research on clinical populations such as autism spectrum disorder (ASD) and social anxiety, where sensitivity to eye contact is often atypical. According to the Fast-Track Modulator Model (Senju and Johnson 2009), disruptions in fast-track gaze processing could contribute to social communication difficulties. In ASD, altered neural responses to direct gaze have been reported, including atypical N170 and P300 modulation depending on predictability (Naples *et al.* 2022) and delayed N170 responses to faces when attention is directed to the eyes (Parker *et al.* 2021). Recent work emphasizes that social cue processing relies on predictive mechanisms that integrate contextual information and prior expectations (Pärnamets and Olsson 2020, Sevgi *et al.* 2020). In this framework, our findings suggest that difficulties may reflect not only reduced sensitivity to gaze cues but also diminished flexibility in adapting gaze responses to context. Embedding eye contact in meaningful, predictable communicative settings may therefore help scaffold gaze processing in these populations.

The present study shows that attentional effects of dynamic gaze are graded rather than automatic. Direct gaze produced stronger N2pc amplitudes than averted gaze, with effects dependent on social task relevance and attention availability. These findings refine gaze processing models, showing that attentional prioritization depends on communicative context and cognitive demands rather than reflexive triggers.

## Conclusion

Across three EEG experiments, we showed that the impact of dynamic gaze on perception and attention is graded rather than automatic. Direct gaze consistently enhanced early perceptual encoding (N170), but its influence on attentional selection (N2pc) varied systematically with task relevance. These findings refine theoretical models of gaze processing by demonstrating that the prioritization of eye contact is not reflexively triggered but flexibly gated by communicative context and attentional demands.

## Supplementary Material

nsag038_Supplementary_Data

## Data Availability

The data supporting the findings of this study are openly available at the Open Science Framework (OSF) at https://osf.io/vbrju/.
