# Supplementary material for: Dynamic gaze processing: robust N170 for direct gaze and task-dependent N2pc effects
Source: Soc Cogn Affect Neurosci. 2026 May 22;21(1):nsag038. doi: 10.1093/scan/nsag038 (PMC13256080; doi:10.1093/scan/nsag038)
Supplement: nsag038_Supplementary_Data [file nsag038_supplementary_data.docx]

**Supplementary Materials**

**Exploratory Brain–Behavior Correlation Analyses**

To examine whether individual differences in gaze-related neural responses were associated with their behavioral counterparts, we conducted exploratory Pearson correlation analyses between condition-level ERP amplitudes (N170 toward, N170 away, N2pc toward, N2pc away) and matched behavioral measures (accuracy and reaction times for the corresponding condition) separately for Experiments 1 and 2 (N = 22 per experiment), and pooled across both experiments (N = 44). A Bonferroni correction was applied to account for 8 comparisons per analysis (corrected α = .006).

**Results**

Results are presented in Tables S1a, S1b, and S1c. Across all analyses, neither N170 nor N2pc amplitudes were reliably correlated with their behavioral counterparts. In Experiment 1, all correlations were non-significant (all ps > .192). In Experiment 2, all correlations likewise remained non-significant (all ps > .054). In the pooled sample, all correlations were non-significant (all ps > .063). No correlation survived the Bonferroni correction in any analysis.

**Table S1a** Pearson Correlations Between Condition-Level ERP Amplitudes and Behavioral Counterparts — Experiment 1

|  | **RT** | **ACC** |
| --- | --- | --- |
| N170 toward | .289 | .003 |
| N170 away | .154 | .062 |
| N2pc toward | .273 | .046 |
| N2pc away | .230 | .118 |

Note. N = 22. df = 20. RT and ACC are matched by condition (toward with toward, away with away). All correlations non-significant (all ps > .192).

**Table S1b** Pearson Correlations Between Condition-Level ERP Amplitudes and Behavioral Counterparts — Experiment 2

|  | **RT** | **ACC** |
| --- | --- | --- |
| N170 toward | .241 | −.218 |
| N170 away | .071 | .362 |
| N2pc toward | .178 | .400 |
| N2pc away | .048 | .417 |

Note. N = 22. df = 20. RT and ACC are matched by condition (toward with toward, away with away). All correlations non-significant (all ps > .054).

**Table S1c** Pearson Correlations Between Condition-Level ERP Amplitudes and Behavioral Counterparts — Experiments 1 and 2 Pooled

|  | **RT** | **ACC** |
| --- | --- | --- |
| N170 toward | .086 | −.005 |
| N170 away | .058 | −.001 |
| N2pc toward | −.166 | .228 |
| N2pc away | −.148 | .232 |

Note. N = 44. df = 42. RT and ACC are matched by condition. All correlations non-significant (all ps > .063; Bonferroni-corrected α = .006).

**Discussion**

These exploratory analyses revealed no significant association between gaze-related ERP amplitudes and their behavioral counterparts in either experiment. Neither N170 nor N2pc amplitudes were significantly correlated with accuracy or reaction times after Bonferroni correction, suggesting that, in the present sample, neural prioritization of gaze cues, whether at the perceptual encoding or attentional selection stage, may operate with a degree of independence from response-level performance. These findings should be interpreted cautiously given the exploratory nature of the analyses and the limited sample size per experiment; future studies with larger samples and pre-registered hypotheses would be needed to draw firmer conclusions.
